# Population Pharmacokinetic Assessment of the Effect of Food on Piperaquine Bioavailability in Patients with Uncomplicated Malaria

**DOI:** 10.1128/AAC.02318-13

**Published:** 2014-04

**Authors:** Joel Tarning, Niklas Lindegardh, Khin Maung Lwin, Anna Annerberg, Lily Kiricharoen, Elizabeth Ashley, Nicholas J. White, François Nosten, Nicholas P. J. Day

**Affiliations:** a Mahidol Oxford Tropical Medicine Research Unit, Faculty of Tropical Medicine, Mahidol University, Bangkok, Thailand; b Centre for Tropical Medicine, Nuffield Department of Medicine, University of Oxford, Oxford, United Kingdom; c Shoklo Malaria Research Unit, Mahidol-Oxford Tropical Medicine Research Unit, Faculty of Tropical Medicine, Mahidol University, Mae Sot, Thailand

## Abstract

Previously published literature reports various impacts of food on the oral bioavailability of piperaquine. The aim of this study was to use a population modeling approach to investigate the impact of concomitant intake of a small amount of food on piperaquine pharmacokinetics. This was an open, randomized comparison of piperaquine pharmacokinetics when administered as a fixed oral formulation once daily for 3 days with (*n* = 15) and without (*n* = 15) concomitant food to patients with uncomplicated Plasmodium falciparum malaria in Thailand. Nonlinear mixed-effects modeling was used to characterize the pharmacokinetics of piperaquine and the influence of concomitant food intake. A modified Monte Carlo mapped power approach was applied to evaluate the relationship between statistical power and various degrees of covariate effect sizes of the given study design. Piperaquine population pharmacokinetics were described well in fasting and fed patients by a three-compartment distribution model with flexible absorption. The final model showed a 25% increase in relative bioavailability per dose occasion during recovery from malaria but demonstrated no clinical impact of concomitant intake of a low-fat meal. Body weight and age were both significant covariates in the final model. The novel power approach concluded that the study was adequately powered to detect a food effect of at least 35%. This modified Monte Carlo mapped power approach may be a useful tool for evaluating the power to detect true covariate effects in mixed-effects modeling and a given study design. A small amount of food does not affect piperaquine absorption significantly in acute malaria.

## INTRODUCTION

Malaria is the most important parasitic disease in humans and kills approximately 660,000 people each year (1). Artemisinin-based combination therapy (ACT) is the recommended first-line treatment worldwide. The artemisinin component is a very potent but short-acting drug and kills the majority of parasites, while the longer-acting partner drug is slowly eliminated and kills residual parasites to prevent recrudescent malaria.

The fixed oral combination of dihydroartemisinin and piperaquine is a promising ACT, which has shown excellent cure rates of 98.7% (95% confidence interval [CI], 97.6 to 99.8%) in a pooled analysis of 3,547 patients with malaria from 12 different sites (2). Dihydroartemisinin-piperaquine is currently recommended as a once-daily dose for 3 days based on patient body weight (daily target dose of 18 mg of piperaquine phosphate/kg of body weight) (3). However, concerns have been raised that small children are underdosed due to the nonlinear relationship between elimination clearance and body weight (4).

Dihydroartemisinin has a very short terminal elimination half-life of approximately 1 h, while piperaquine has a long half-life of approximately 20 to 30 days (4–7). The absolute oral bioavailability of piperaquine has not been reported in humans, since parenteral formulations are unavailable. However, a 50% oral bioavailability compared to an experimental parenteral formulation was reported in rats (8). Piperaquine is a highly lipophilic drug, and its absorption might be facilitated by concomitant intake of fat, as described previously for other lipid-soluble antimalarial drugs such as lumefantrine (9), halofantrine (10), mefloquine (11), and atovaquone (12).

Contradictory results have been reported for the effects of concomitant food intake on piperaquine pharmacokinetics in healthy volunteers and patients with malaria. Sim and colleagues reported a 98% increase in total exposure of piperaquine after a high-fat breakfast (53 g fat) in healthy Caucasian volunteers (*n* = 8; crossover design) (13). Nguyen et al. reported a more modest 41% increase in total piperaquine exposure after a standardized Vietnamese breakfast (17 g fat) in healthy Vietnamese volunteers (*n* = 14; parallel design) (14). However, Hai and colleagues reported no significant impact on piperaquine pharmacokinetics after a similar standardized Vietnamese breakfast (17 g fat) in healthy Vietnamese volunteers (*n* = 32; parallel design) (15). The noncompartmental analysis of data presented here showed no effect of concomitant intake of a small amount of fat (6.4 g fat) on piperaquine exposure in patients with uncomplicated Plasmodium falciparum malaria in Thailand (*n* = 30; parallel design) (16). However, all reported studies used a noncompartmental approach with a low statistical power for detecting concomitant food intake as an influential factor on piperaquine pharmacokinetics, compared to a modeling approach.

The aim of this study was to use a potentially more powerful population approach to analyze data from a previously reported study (16) to evaluate the effects of concomitant food intake on the pharmacokinetic properties of piperaquine in patients with uncomplicated P. falciparum malaria in Thailand.

## MATERIALS AND METHODS

### Study design.

This was an open, randomized, parallel study of piperaquine pharmacokinetics when administered as a fixed oral formulation once daily for 3 days with and without concomitant fat to patients with uncomplicated P. falciparum malaria in Thailand. Clinical and noncompartmental results were reported in full previously (16). Briefly, 30 patients aged 16 to 65 years were enrolled and randomized into one of the two treatment arms. Inclusion criteria were microscopy confirmation of asexual P. falciparum or mixed infections, no signs of severe malaria, and willingness to participate in the study (fully informed consent). Exclusion criteria were ≥4% red blood cell parasitemia, positive urine test for pregnancy, known hypersensitivity to artemisinins or piperaquine, treatment with dihydroartemisinin or piperaquine within the past 4 months, or hematocrit level of <30%. Full demographics are given in Table 1. Ethical approval was granted by the Faculty of Tropical Medicine Mahidol University Ethical Committee, Bangkok, Thailand, and the Oxford Tropical Research Ethics Committee (OxTREC), United Kingdom.

**TABLE 1:** Demographics of patients with uncomplicated P. falciparum malaria in Thailand

Parameter	Value for group	
	Fasting patients	Fed patients
Pharmacokinetic data		
Total no. of patients	15	15
Total no. of piperaquine samples	535	541
Median daily dose of piperaquine phosphate (mg/kg) (range)	17.2 (16.0–18.6)	17.5 (16.0–18.8)
Median daily dose of dihydroartemisinin (mg/kg) (range)	2.14 (2.00–2.33)	2.19 (2.00–2.35)
Demographics		
Median age (yr) (range)	38 (18–55)	28 (19–45)
Median body wt (kg) (range)	50 (39–62)	53 (45–73)
No. of males/no. of females	13/2	13/2
Median axillary temp at admission (°C) (range)	36.5 (36.2–38.2)	37.1 (35.9–39.2)
Median parasitemia at admission (no. of parasites/μl) (range)	8,000 (448–140,000)	8,000 (352–60,000)
Median diastolic blood pressure (mmHg) (range)	70 (60–80)	70 (60–110)
Median systolic blood pressure (mmHg) (range)	110 (90–130)	110 (90–140)
Median hematocrit (%) (range)	41 (30–45)	42 (33–47)
Median pulse (beats/min) (range)	80 (65–96)	84 (72–120)

### Drug administration.

All patients were given a supervised standard fixed oral formulation of dihydroartemisinin and piperaquine (40 mg dihydroartemisinin and 320 mg piperaquine phosphate per tablet) (Duo-Cotecxin; Beijing Holley-Cotec Pharmaceuticals Co., Ltd., China). A weight-based dose regimen, once-daily treatment for 3 days, was employed to achieve a daily target dose of 18 mg of piperaquine phosphate/kg of body weight (Table 1). All patients in the fed group (*n* = 15) received a 200-ml carton of chocolate milk, containing 6.4 g of fat, with each dose. Patients in the fasting group (*n* = 15) were given study medication at enrollment, after at least 2 h of fasting, and consecutive doses after an overnight fast. Patients were asked to continue fasting for 3 h after each dose.

### Pharmacokinetic sampling.

Blood samples during the intensive collection phase (up to 12 h after the last dose) were collected through an indwelling intravenous cannula flushed with 0.5 ml heparinized saline solution after each sample collection. A total of 0.5 ml of blood was discarded immediately before sampling, and blood samples (2 to 5 ml) were collected into lithium heparin tubes predose (0 h); at 0.5, 1, 2, 3, 4, 7, and 24 h after the first dose; at 1, 3, 4, 5, 7, and 24 h after the second dose; and at 1, 2, 3, 4, 5, 6, 8, and 12 h after the third dose. Additional samples were collected by venipuncture on days 4, 5, 7, 14, 21, 28, 42, 56, 70, 84, 98, 112, and 126 after the first dose. All blood samples were centrifuged at 2,000 × *g* for 10 min, and plasma was stored in liquid nitrogen within 30 min of collection. Plasma samples were transported on dry ice to the Department of Clinical Pharmacology at the Mahidol-Oxford Tropical Medicine Unit, Faculty of Tropical Medicine, Mahidol University, Bangkok, Thailand. Piperaquine plasma samples were quantified by using a previously reported high-throughput assay consisting of liquid chromatography linked with tandem mass spectrometry detection (17). Triplicates of quality control samples at three different concentrations (i.e., 4.5, 20, and 400 ng/ml) were analyzed within each batch of samples to ensure precision and accuracy during drug measurements. The coefficient of variation was below 5% for all quality control samples. The lower limit of quantification was 1.2 ng/ml.

### Population pharmacokinetics.

Piperaquine plasma concentrations were transformed into their natural logarithms and evaluated by using nonlinear mixed-effects modeling in NONMEM v.7.2 (Icon Development Solutions, MD). Automation and visualization of diagnostic results were performed by using Pearl-Speaks-NONMEM (PsN) v.3.6.2 (18, 19), Xpose v.4 (20), R v.2.13.1 (R Foundation for Statistical Computing), and Pirana v.2.6 (21).

Subroutine ADVAN5 and the first-order conditional estimation method with interactions were used throughout modeling. The objective function value (OFV) computed by NONMEM as minus twice the log likelihood of data, goodness-of-fit diagnostics, and simulation-based diagnostics were used to discriminate between models. A total of 91 out of 1,016 samples (9.1%) were quantified below the limit of quantification and omitted during model building. The majority of these omitted samples (82.6%) were in the late terminal phase (>70 days) during the long follow-up period. The impact of omitting these samples was evaluated by using the final model.

One-, two-, three-, and four-compartment distribution models were first evaluated during model building. The best-performing distribution model was assessed together with different absorption models (i.e., first-order absorption with and without lag time and a more flexible transit compartment absorption with a fixed number of transit compartments for the population [22]). Implementation of a fixed relative bioavailability of 100% for the population but allowing for between-subject variability and between-dose occasion variability (i.e., modeled as within-subject variability between doses) in the same parameter was also evaluated. Pharmacokinetic parameters were modeled assuming log-normal distributions, while between-subject variability and between-dose occasion variability were assumed to be normally distributed with a zero mean and ω^2^ variance. The random residual variability was assumed to be additive, since data were modeled as natural logarithms (i.e., essentially equivalent to an exponential error model for untransformed data).

Body weight was investigated as a covariate by using an allometric function with power values of 3/4 for clearance parameters and 1 for volume parameters. Systematic variability in the relative bioavailability between dose occasions was evaluated as a categorical and a linear covariate for doses 1, 2, and 3. Effects of age, initial parasitemia, hematocrit, temperature upon admission, sex, and concomitant food intake on all parameters were evaluated formally with a stepwise forward-addition (*P* < 0.05) and backward-deletion (*P* < 0.01) covariate approach by using the automated Stepwise Covariate Model (SCM) implemented in PsN. Continuous covariates were tried as linear, piecewise linear, power, and exponential functions, and categorical covariates were tried as linear functions. The final structural model, including body weight as an allometric function on clearance and volume parameters as well as between-subject variability on all parameters and between-dose occasion variability on relative bioavailability and mean absorption time, was also used to investigate the impact of concomitant fat intake on absorption parameters with a full covariate approach (23). Concomitant fat intake was simultaneously implemented as a categorical food effect on the relative bioavailability and absorption parameters, followed by bootstrap diagnostics (*n* = 500) to evaluate the clinical relevance of such potential covariate effects.

Basic goodness-of-fit diagnostics, visual and numerical predictive checks, as well as bootstrap diagnostics were used to evaluate the appropriateness of the final model. Visual predictive checks were visualized by plotting the 95% confidence intervals of the 5th, 50th, and 95th simulated (*n* = 2,000) percentiles against measured piperaquine concentrations. The nonparametric bootstrap diagnostics (*n* = 1,000) were stratified by drug administration with or without food to maintain an equal distribution of patients in the resampled data.

### Evaluation of population versus noncompartmental approaches.

A traditional power calculation was performed by using the mean results (± standard deviations [SD]) of total piperaquine exposure from the noncompartmental analysis reported previously (16). The mean total piperaquine exposure in fasting patients was used to calculate the mean exposure in fed patients after a putative food effect, and the SD was based on that observed for fasting patients but was assumed to be proportional to exposure. Standard functionalities in STATA v.12.0 (Stata Corp., TX, USA) were used to calculate the minimum effect size of food needed for a statistically significant difference between a fasting group (*n* = 15) and a fed group (*n* = 15) (power = 0.8; alpha = 0.05).

An embedded functionality in PsN for rapid sample size calculations of mixed-effects models (24) was modified to calculate the minimum effect size of food on relative bioavailability needed for a statistically significant covariate relationship (modified Monte Carlo mapped power approach). The original study design of 15 fed and 15 fasting patients was extended to include 11,000 simulated patients (50% in the fed group and 50% in the fasting group). Individual concentration-time data were simulated for these patients with the final model with the addition of a fixed categorical effect size of 5% to 50% of food on relative bioavailability (i.e., 1,000 patients for each effect size with an increment of 5% between simulations). The simulated data were then separately reestimated for each effect size with the final model with and without a food effect on relative bioavailability. The difference in individual objective function values (ΔiOFV) was calculated for each patient for the two models. Fifteen ΔiOFVs were resampled at random (bootstrap; *n* = 10,000) from each group (i.e., fed and fasting) and effect size (i.e., 5% to 50%), and fractions of the 10,000 bootstrap samples above the critical total OFV (ΣΔiOFV) of 3.84 and 6.63 (i.e., power at alpha values of 0.05 and 0.01, respectively) were plotted against the evaluated covariate effect sizes.

## RESULTS

The study medication was well tolerated, with no severe adverse reactions reported during the study period. There were no statistical differences between the two groups in terms of demographic variables (Table 1). A total of eight patients had recurrent malaria during the 126 days of follow-up, but none of these patients were classified as having recrudescent malaria by PCR genotyping. The median time to a new infection was 42 days (range, 32 to 87 days) in the fasting group (*n* = 5) and 70 days (range, 58 to 125 days) in the fed group (*n* = 3).

### Population pharmacokinetics.

**FIG 1:**
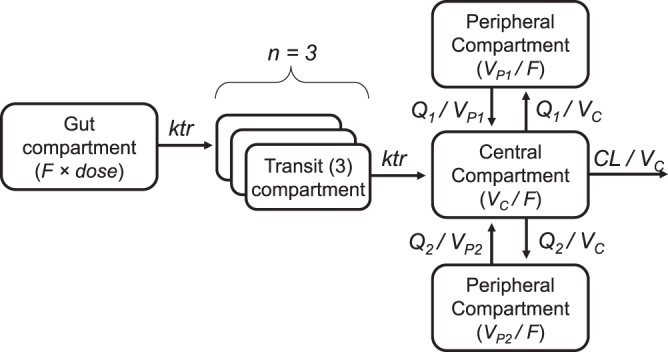
Final structural model for piperaquine population pharmacokinetics in fasting (*n* = 15) and fed (*n* = 15) patients with uncomplicated P. falciparum malaria in Thailand. CL, elimination clearance; *F*, relative oral bioavailability; *ktr*, transit absorption rate constant; *Q*, intercompartment clearances; *V_C_*, apparent volume of distribution of the central compartment; *V_P_*, apparent volume of distribution of the peripheral compartments; MTT, mean absorption transit time; *n*, number of transit compartments [MTT = (*n* + 1)/*ktr*].

Piperaquine population pharmacokinetics were well described by a 3-compartment distribution model, with no significant improvement by adding an additional peripheral distribution compartment (ΔOFV = −5.08). A transit compartment (*n* = 3) absorption model described the absorption phase well and was superior to all other absorption models (ΔOFV < −51.9). The absorption rate from the last transit compartment could be set as identical to the rate constant between transit compartments without a significant impact on the model (ΔOFV = 0.41). This also provided a more stable absorption model. The final structural model is shown in Fig. 1.

Implementation of a fixed relative bioavailability of 100% for the population but allowing for between-subject variability and between-dose occasion variability in the same parameter improved the model fit significantly (ΔOFVs of −46.1 and −160, respectively, when implemented sequentially). However, the between-subject variability could be removed after implementation of between-dose occasion variability without a major impact on the model fit (ΔOFV = 1.94). Between-dose occasion variability in the mean absorption time also improved the model significantly (ΔOFV = −27.6).

**FIG 2:**
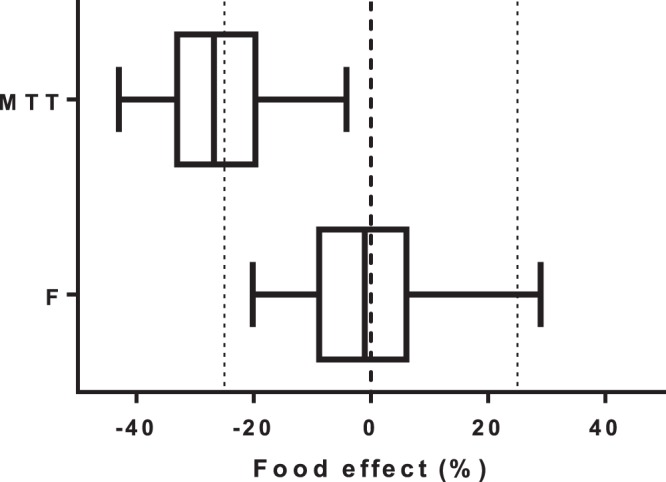
Box plots (interquartile ranges with 2.5 to 97.5 percentiles) showing the effect of estimated food effects on mean absorption transit time (MTT) and relative bioavailability (*F*). Vertical dashed lines indicate no effect and ±25% effects.

A fixed allometric function for body weight improved the model fit marginally (ΔOFV = −3.68) but was kept in the final model based on prior strong physiological evidence for such a covariate relationship. Dose-occasion as a categorical covariate effect on relative bioavailability improved the model significantly (ΔOFV = −11.0) and resulted in 21.9% and 50.9% increased bioavailability at dose 2 and dose 3, respectively, compared to dose 1. However, this could be simplified to a linear covariate relationship (i.e., 25.3% increase in bioavailability per dose) without a significant reduction in model fit (ΔOFV = −0.062). Effects of age and sex on peripheral distribution volume and concomitant food intake on mean absorption time were significant covariates in the forward-addition step (*P* < 0.05), but only the effect of age on the peripheral volume of distribution could be retained in the backward-elimination step with a more parsimonious cutoff (*P* < 0.01). This covariate relationship resulted in a linear increase in the peripheral volume of distribution of 4.10% for each year of age increase. A separate bootstrap diagnostic from the implementation of a food effect on mean absorption time and relative bioavailability showed a significantly higher absorption rate during concomitant food intake but no effect on total bioavailability (Fig. 2).

**FIG 3:**
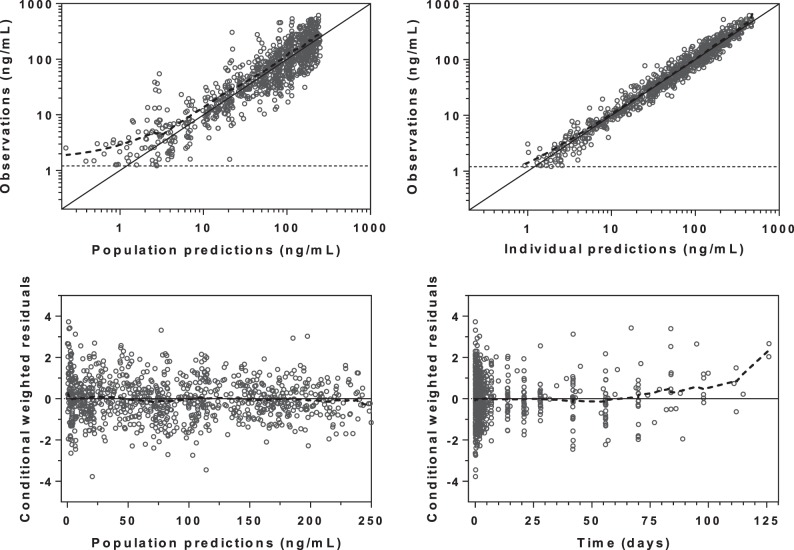
Goodness-of-fit diagnostics of the final population pharmacokinetic model of piperaquine in fasting (*n* = 15) and fed (*n* = 15) patients with uncomplicated P. falciparum malaria. Broken lines, locally weighted least-squares regression; solid lines, line of identity; broken horizontal lines, lower limit of quantification. The observed concentrations, population predictions, and individual predictions were transformed into their logarithms (base 10).

**FIG 4:**
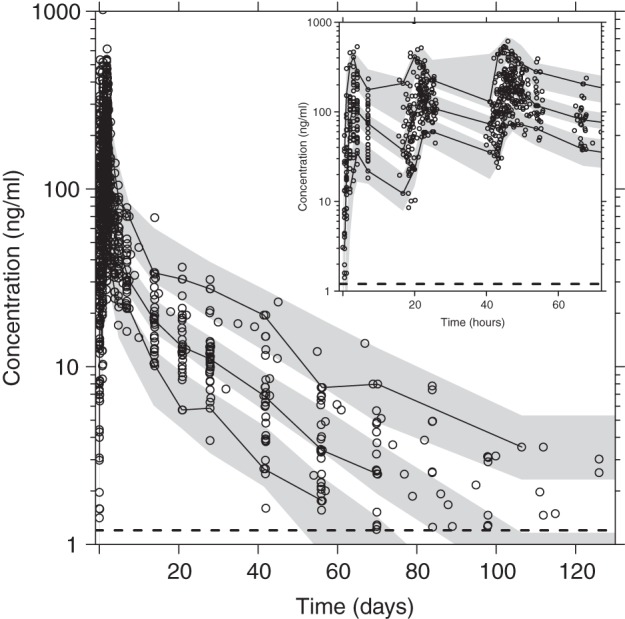
Visual predictive check of the final model describing the population pharmacokinetics of piperaquine in fasting (*n* = 15) and fed (*n* = 15) patients with uncomplicated P. falciparum malaria. The inset shows piperaquine simulations at 0 to 72 h. Open circles, observed data points; solid lines, 5th, 50th, and 95th percentiles of the observed data; shaded areas, 95% confidence intervals of simulated (*n* = 2,000) 5th, 50th, and 95th percentiles. Broken horizontal lines are the lower limit of quantification. Venous plasma piperaquine concentrations were transformed into their logarithms (base 10).

Basic goodness-of-fit diagnostics resulted in adequate model performance, with no obvious model misspecification (Fig. 3). However, a minor trend of data censoring at the lower limit of quantification could be seen, but this resulted in no model misspecification in terms of simulated and observed fractions of censored data (data not shown). Furthermore, omitting data below the limit of quantification resulted in almost identical parameter estimates as when these were handled as categorical data (i.e., the M3 method resulted in a <5.5% absolute mean bias compared to when the data were omitted) (25). The predictive checks demonstrated excellent predictive performance of the final model (Fig. 4) and calculated 4.32% (95% CI, 2.38 to 8.43%) and 5.62% (95% CI, 2.27 to 8.32%) of the observed data below and above the simulated 90% prediction interval. Final parameter estimates and bootstrap diagnostics are summarized in Tables 2 and 3.

**TABLE 2:** Population estimates of the final model describing piperaquine population pharmacokinetics in fasting (*n* = 15) and fed (*n* = 15) patients with uncomplicated P. falciparum malaria in Thailand

Parameter^*d*^	Value			
	Population estimate^*a*^ (% RSE^*b*^)	95% CI for population estimate^*b*^	IIV (% CV)^*a*^ (% RSE^*b*^)	95% CI for IIV^*b*^
Typical parameters				
CL/*F* (liters/h)	67.6 (11.6)	54.0–85.5	24.4 (26.0)	17.4–29.6
*V_C_*/*F* (liters)	3,030 (16.4)	2,160–4,180	51.6 (32.3)	31.2–68.1
*Q*_1_/*F* (liters/h)	408 (15.0)	309–557		
*V_P_*_1_/*F* (liters)	6,240 (14.6)	4,890–8,530	45.6 (48.8)	18.8–68.4
*Q*_2_/*F* (liters/h)	109 (13.6)	83.3–143	25.8 (48.2)	6.67–37.9
*V_P_*_2_/*F* (liters)	24,400 (10.1)	20,000–29,500		
MTT (h)	2.04 (7.50)	1.80–2.41	24.1 (52.7)/39.4 (22.7)^*c*^	8.77–35.6/29.2–48.0
No. of transit comp.	3 (fixed)			
*F* (%)	100 (fixed)		48.8 (16.6)^*c*^	38.3–56.0
σ (% CV)	30.7 (4.42)	27.8–33.5		
Covariate effects				
Dose effect on *F* (%)	25.3 (34.4)	9.82–53.2		
Age effect on *V_P_*_1_ (%)	4.10 (18.1)	2.38–5.32		

a Computed population mean values from NONMEM. Interindividual variability (IIV), between-occasion variability, and random residual variability are calculated as 100 × $\sqrt{EXP(\sigma^{2})-1}$.

b Assessed by the nonparametric bootstrap method (*n* = 1,000 iterations) for the final pharmacokinetic model. Relative standard errors (RSE) are calculated as 100 × (standard error/mean). Ninety-five-percent confidence intervals are displayed as the 2.5 to 97.5 percentiles of bootstrap estimates.

c Between-occasion variability.

d CL, elimination clearance; *V_C_*, central volume of distribution; *Q*, intercompartment clearance; *V_P_*, peripheral volume of distribution; MTT, mean absorption transit time; No. of transit comp., number of transit compartments; *F*, oral bioavailability; σ, additive residual error; CV, coefficient of variation.

**TABLE 3:** *Post hoc* estimates of the final model describing piperaquine population pharmacokinetics in fasting (*n* = 15) and fed (*n* = 15) patients with uncomplicated P. falciparum malaria in Thailand

Parameter for *post hoc* estimate^*b*^	Median value for group (IQR)^*a*^			*P* value
	Total	Fasting patients	Fed patients	
CL/*F* (liters/h/kg)	1.02 (0.802–1.21)	1.09 (0.745–1.31)	0.988 (0.867–1.10)	0.548
*V*/*F* (liters/kg)	516 (378–613)	488 (375–607)	517 (411–594)	0.917
*t*_1/2_ (days)	19.5 (16.8–21.8)	19.8 (16.2–21.3)	19.1 (18.1–21.8)	0.520
*C*_max_ (ng/ml)	237 (169–347)	242 (165–319)	231 (177–395)	0.663
*T*_max_ (h)	3.57 (3.08–4.27)	3.36 (2.80–4.20)	3.64 (3.25–4.16)	0.520
AUC_inf_ (h × μg/ml)	26.8 (22.7–34.2)	23.9 (21.6–36.7)	27.5 (25.2–32.7)	0.419
Day 7 concn (ng/ml)	29.8 (26.1–35.4)	26.9 (22.1–42.7)	30.9 (28.3–34.3)	0.395

a *Post hoc* estimates were calculated as median values with interquartile ranges (IQR) from empirical Bayes estimates, and statistical differences were estimated with a nonparametric Mann-Whitney test.

b CL, elimination clearance; *F*, oral bioavailability; *V*, apparent total volume of distribution (*V_C_* + *V_P1_* + *V_P2_*); *t*_1/2_, terminal elimination half-life; AUC_inf_, area under the concentration-time curve from time point 0 to day 138; *C*_max_, predicted peak concentration; *T*_max_, predicted time to peak concentration; Day 7 concn, predicted concentration at day 7.

### Evaluation of population versus noncompartmental approaches.

**FIG 5:**
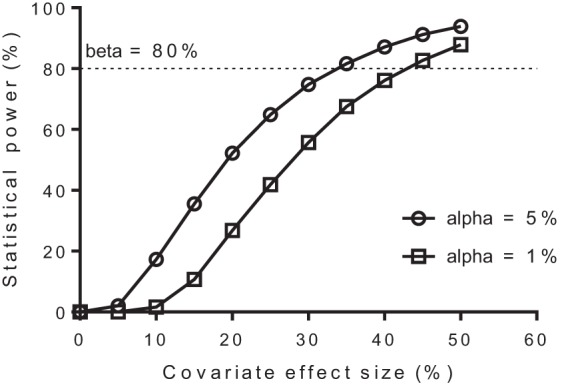
Modified Monte Carlo mapped power approach, showing the statistical power to detect various degrees of covariate effects with the final pharmacokinetic model and the given study design (15 fed and 15 fasting patients).

A noncompartmental analysis and a groupwise statistical comparison of total piperaquine exposure between the fed and fasting groups resulted in 80% power (alpha = 0.05) to detect a minimum difference of 81%. However, the novel power methodology for mixed-effects models resulted in 80% power (alpha = 0.05) for a covariate effect of 35%, thus proving the study design to be adequately powered for a population approach if a putative clinically relevant difference of >35% would be present between fed and fasting patients. The statistical power to detect various degrees of covariate effects is illustrated in Fig. 5.

## DISCUSSION

The fixed oral combination of dihydroartemisinin and piperaquine has demonstrated excellent cure rates in adult patients with P. falciparum malaria in Thailand (2). In this small study, there was no recrudescent malaria when the combination was administered alone or together with a standardized meal. The relatively high rate of new infections is most likely a consequence of the long follow-up period of 125 days.

### Population pharmacokinetics.

The population pharmacokinetics of piperaquine were well characterized by the study design and the developed nonlinear mixed-effects model. Piperaquine showed a multiphasic distribution, as described previously (4, 7, 26). A more flexible absorption model improved the model significantly and described the variable absorption of piperaquine satisfactorily. A major improvement was seen when allowing for between-dose occasion variability, which confirms the large variability in the absorption of piperaquine both between and within subjects. The estimated relative bioavailability of piperaquine increased by 25.3% for each consecutive dose. This was not related to concomitant food intake and might be a consequence of patients recovering from malaria during the course of the treatment and an improvement in general clinical status. This phenomenon has been described previously for piperaquine treatment in pregnant women (7) as well as for mefloquine treatment in children with acute uncomplicated P. falciparum malaria (27). The peripheral volume of distribution increased with age in this study, which is likely to reflect the increased body fat-to-water content in older patients. However, this is unlikely to have clinical implications considering that total exposure of piperaquine was not affected. Final pharmacokinetic parameter estimates were generally in good agreement with values reported for adult patients with malaria (5, 7).

This study demonstrated no clinical impact on concomitant intake of a small amount of fat on the total absorption, day 7 levels, peak concentrations, or time to peak concentrations of piperaquine. Previous studies in healthy volunteers reported a large impact on the relative bioavailability of piperaquine after a high-fat meal (13, 14). These differences are likely to be explained by the small amount of fat administered in this study, but we believe that it reflects the clinical reality, as many patients are unable to consume a full meal when presenting with malaria. Most patients are unwell when presenting with malaria, and nausea is commonly seen in these patients. One carton of milk seems a reasonable maximum amount of fat that patients would be likely to consume during acute illness and treatment.

A 26% higher mean absorption rate of piperaquine (95% CI, 5.1 to 42%) was seen in patients who received study treatment with concomitant food as compared to fasting patients when using a full covariate approach. This is most likely a result of the rapid gastric emptying associated with consumption of liquids (28) and/or the increased dissolution of piperaquine when coadministered with fatty liquids. However, dihydroartemisinin is primarily responsible for the initial parasite-killing effect, and this trend of an increased absorption rate of piperaquine is not likely to have any substantial clinical impact with respect to treatment outcome. Indeed, the prophylactic protective effect of dihydroartemisinin-piperaquine in volunteers (*n* = 800) at risk of malaria infections in Thailand was not significantly different when administered with or without food (29). Furthermore, the trend of an increased absorption rate associated with concomitant food intake did not result in higher predicted peak levels of piperaquine in the final model, so it is not likely to have any clinical impact. This suggests that the manufacturer's recommendation of taking the treatment without food (up to 3 h after the dose) could be reviewed.

### Evaluation of population versus noncompartmental approaches.

The modified Monte Carlo mapped power approach was implemented successfully and enabled a numerical approach to evaluate the power to detect various degrees of food effects with the given study design. It demonstrated a marked increase in statistical power when using a population approach compared to a noncompartmental analysis with a traditional groupwise statistical comparison (35% versus 81% difference needed for statistical significance with the competing approaches). This evaluation also lends further support to the results, since it demonstrated that the study was adequately powered to detect clinically relevant pharmacokinetic differences. A population approach also offers other advantages, such as a mechanistic understanding of the pharmacokinetics and influential covariates as well as a quantitative measure of the variability between and within patients. However, it is of course more time-consuming than a traditional noncompartmental approach. Small pharmacokinetic studies are often a reality in antimalarial drug research, and the presented novel power approach could be a useful tool to evaluate the power of detecting true covariate effects in mixed-effects modeling of a given study design.

In conclusion, piperaquine population pharmacokinetics were well described in fasting and fed patients with uncomplicated P. falciparum malaria in Thailand by a three-compartment distribution model with a flexible absorption model. The final model showed a marked increase in bioavailability during recovery from malaria but demonstrated no clinically significant impact of concomitant intake of a low-fat meal. The novel methodology for assessing the power of detecting a true covariate relationship presented may be used in mixed-effects modeling to provide a numerical approach to conclude a lack of covariate effects.
